# Na^+^/H^+^-exchanger-1 inhibition counteracts diabetic cataract formation and retinal oxidative-nitrative stress and apoptosis

**DOI:** 10.3892/ijmm.2012.933

**Published:** 2012-03-07

**Authors:** SERGEY LUPACHYK, ROMAN STAVNIICHUK, JULIA I. KOMISSARENKO, VIKTOR R. DREL, ALEXANDER A. OBROSOV, AZZA B. EL-REMESSY, PAL PACHER, IRINA G. OBROSOVA

**Affiliations:** 1 Pennington Biomedical Research Center, Louisiana State University System, Baton Rouge, LA, USA; 2 Department of Endocrinology, National Medical University, Kiev, Ukraine; 3 College of Pharmacy, University of Georgia, Augusta, GA, USA; 4 Section on Oxidative Stress Tissue Injury, Laboratory of Physiological Studies, NIH/NIAAA, Bethesda, MD, USA

**Keywords:** apoptosis, cariporide, diabetic cataract, early diabetic retinopathy, retinal endothelial cell, Na^+^/H^+^-exchanger-1, oxidative-nitrative stress, retinal pericyte

## Abstract

The Na^+^-H^+^-exchanger-1 (NHE-1) controls intracellular pH and glycolytic enzyme activities, and its expression and activity are increased by diabetes and high glucose. NHE-1-dependent upregulation of the upper part of glycolysis, under conditions of inhibition (lens) or insufficient activation (retina) of glyceraldehyde 3-phosphate dehydrogenase, underlies diversion of the excessive glycolytic flux towards several pathways contributing to oxidative stress, a causative factor in diabetic cataractogenesis and retinopathy. This study evaluated the role for NHE-1 in diabetic cataract formation and retinal oxidative stress and apoptosis. Control and streptozotocin-diabetic rats were maintained with or without treatment with the NHE-1 inhibitor cariporide (Sanofi-Aventis, 10 mgkg^−1^d^−1^) for 3.5 months. In *in vitro* studies, bovine retinal pericytes and endothelial cells were cultured in 5 or 30 mM glucose, with or without 10 μM cariporide, for 7 days. A several-fold increase of the by-product of glycolysis, α-glycerophosphate, indicative of activation of the upper part of glycolysis, was present in both rat lens and retina at an early (1-month) stage of streptozotocin-diabetes. Cariporide did not affect diabetic hyperglycemia and counteracted lens oxidative-nitrative stress and p38 MAPK activation, without affecting glucose or sorbitol pathway intermediate accumulation. Cataract formation (indirect ophthalmoscopy and slit-lamp examination) was delayed, but not prevented. The number of TUNEL-positive cells per flat-mounted retina was increased 4.4-fold in diabetic rats (101±17 vs. 23±8 in controls, P<0.01), and this increase was attenuated by cariporide (45±12, P<0.01). Nitrotyrosine and poly(ADP-ribose) fluorescence and percentage of TUNEL-positive cells were increased in pericytes and endothelial cells cultured in 30 mM glucose, and these changes were at least partially prevented by cariporide. In conclusion, NHE-1 contributes to diabetic cataract formation, and retinal oxidative-nitrative stress and apoptosis. The findings identify a new therapeutic target for diabetic ocular complications.

## Introduction

Diabetic retinopathy (DR) and diabetic cataract (DC) are two chronic ocular complications of diabetes mellitus associated with vision loss. Vision-threatening DR is present is one out of twelve diabetic subjects in the age group of 40 years and older (1). The predominant cause of visual loss in DR is a clinically significant macular edema or proliferative DR associated with neovascularization which results in tractional retinal detachment or non-clearing vitreous hemorrhage (2,3). A cataract is a clouding of the natural lens, the part of the eye responsible for focusing light and producing clear, sharp images. Several large epidemiological studies revealed up to 5-fold increased prevalence of cataracts with cortical and/or posterior subcapsular opacities in diabetic subjects compared with the non-diabetic population (4–8). In the Wisconsin Epidemiologic Study of Diabetic Retinopathy (9), cataract was identified as a significant cause of legal blindness, second only to proliferative diabetic retinopathy, in younger onset diabetic subjects, and the most frequent cause of visual loss in older onset diabetic subjects.

Multiple mechanisms including, but not limited to, increased sorbitol pathway activity (1,10), activation of the advanced glycation end-product (AGE)/receptor for the advanced glycation end-product (RAGE) axis (11,12) and poly(ADP-ribose) polymerase (13,14), and proinflammatory response (15–18), have been implicated in the pathogenesis of both DR and DC. Growing evidence suggests that both these and other [activation of protein kinase C (19,20), cyclooxygenase (21), 12/15-lipoxygenase (22), p38 mitogen-activated protein kinase (23)] metabolic imbalances contributing to diabetes-induced end-organ damage converge at the level of oxidative-nitrative stress. Clinical trials aimed at inhibiting several of the afore-mentioned mechanisms did not yield a pathogenetic treatment for DR or effective anti-cataract agent. Identification of new therapeutic targets to effectively combat oxidative-nitrative stress and diabetic ocular complications is, therefore, highly warranted.

Diabetes-induced upregulation of the upper part of glycolysis, under conditions of inhibition (lens) or insufficient activation (retina) of glyceraldehyde 3-phosphate dehydrogenase, underlies diversion of the excessive glycolytic flux towards formation of methylglyoxal and α-glycerophosphate, with concomitant NAD^+^/NADH redox imbalances and AGE/RAGE axis and NAD(P)H oxidase activation thus leading to enhanced oxidative-nitrative stress (24–26). The mechanisms underlying diabetes-associated activation of the upper part of glycolysis remain unidentified. The Na^+^/H^+^-exchanger-1 (NHE-1), an isoform of NHE ubiquitously distributed in mammalian tissues, plays a pivotal role in the regulation of intracellular pH by removing intracellular protons in exchange for extracellular sodium (27). Upregulation of NHE-1 leads to cytosol alkalinization with resultant activation of glucose transport (28) and glycolytic enzymes including hexokinase (28), hexose phosphate isomerase (29), phosphofructokinase (30), and aldolase (31). Phosphofructokinase is particularly sensitive to NHE-mediated change in intracellular pH (32). Under normal conditions, a ~0.3 unit increase in intracellular pH caused one order of magnitude increase in the rate of glycolysis (33). Evidence for overexpression and activation of NHE-1 in cell and tissue targets for diabetic complications, including retina, is emerging (34–36). It has, therefore, been hypothesized that NHE-1 plays a major role in diabetes-associated upregulation of the upper part of glycolysis in the lens and retina, and that NHE-1 inhibition will counteract oxidative-nitrative stress and both ocular complications. In the present study, this hypothesis has been tested in animal and cell culture experiments with the potent and specific NHE-1 inhibitor, cariporide.

## Materials and methods

### Reagents

Unless otherwise stated, all chemicals were of reagent-grade quality, and were purchased from the Sigma Chemical Co. (St. Louis, MO). Cariporide [N-(diamino-methylidene)-3-methanesulfonyl-4-(propan-2-yl)benzamide] was obtained from Sanofi-Aventis (Frankfurt, Germany). Rabbit polyclonal anti-nitrotyrosine (NT) antibody was purchased from Upstate (Lake Placid, NY), and mouse monoclonal anti-poly(ADP-ribose) antibody from Trevigen, Inc. (Gaithersburg, MD). Rabbit polyclonal antibodies against phosphorylated p38 mitogen-activated protein kinase (MAPK) and phosphorylated extracellular signal-regulated kinase (ERK) were obtained from Cell Signaling Technology, Boston, MA. Rabbit polyclonal antibody antibody against total p38 MAPK, and mouse monoclonal antibody against total ERK were purchased from Santa Cruz Biotechnology, Inc. (Santa Cruz, CA). Secondary Alexa Fluor 488 goat anti-mouse and Alexa Fluor 488 goat anti-rabbit antibodies, Prolong Gold Antifade Reagent, and 4′,6-diamidino-2-phenylindole (DAPI) were purchased from Invitrogen (Eugene, OR). ApopTag^®^ Plus Fluorescein In Situ Apoptosis Detection kit and ApopTag^®^ Peroxidase In Situ Apoptosis Detection kit were purchased from Chemicon International, Inc. (Temecula, CA). Micromount mounting medium was purchased from Surgipath Medical Industries (Richmond, IL). Other reagents for immunohistochemistry were purchased from Dako Laboratories, Inc. (Santa Barbara, CA).

### Animals

The experiments were performed in accordance with regulations specified by the Guide for the Care and Handling of Laboratory Animals (NIH Publication no. 85–23) and Pennington Biomedical Research Center Protocol for Animal Studies. Male Wistar rats (Charles River, Wilmington, MA), body weight 250–300 g, were fed a standard rat chow (PMI Nutrition International, Brentwood, MO) and had access to water *ad libitum*. Streptozotocin (STZ)-diabetes was induced as described (37). Blood samples for glucose measurements were taken from the tail vein ~48 h after the STZ injection and the day prior to the study termination. All rats with blood glucose levels >13.8 mM were considered diabetic. The experimental groups comprised control and diabetic rats treated with or without the NHE-1 inhibitor, cariporide, 10 mgkg^−1^d^−1^, for 3.5 months starting from induction of diabetes. Lens changes were evaluated by an indirect ophthalmoscope and a portable slit lamp (Kowa Co., Tokyo, Japan) weekly. Evaluations were preceded by mydriasis with topical 1% tropicamide hydrochloride. Cataracts were scored as follows: 1, no cataract (clear lenses); 2, equatorial vacuoles; 3, cortical opacities; and 4, mature cataract when the whole lens becomes opaque. The average cataract score was calculated for each rat weekly. Part of the control rats and rats with 1-month duration of STZ-diabetes were used for assessment of lens glucose, sorbitol pathway intermediates, α-glycerophosphate, 4-hydroxynonenal (HNE) protein adducts, nitrotyrosine (NT), total and phosphorylated p38 mitogen-activated protein kinase (MAPK) and extracellular-signal-regulated kinase (ERK), as well as retinal α-glycerophosphate.

### Anesthesia, euthanasia and tissue sampling

The animals were sedated by CO_2_, and immediately sacrificed by cervical dislocation. One eye from several control rats and rats with 3.5-month duration of diabetes was enucleated and fixed in normal buffered 4% formalin. Several retinal sections from the two aforementioned groups were used for obtaining representative pictures of apoptotic nuclei using the ApopTag^®^ Plus Fluorescein In Situ Apoptosis Detection kit. The rest of the eyes were fixed in 4% paraformaldehyde in phosphate-buffered saline (PBS) for preparation of flat mounted retinas and quantitation of apoptosis.

### Specific methods employed in animal studies

#### Immunohistochemical studies

All flat-mounted retinas were processed by a single investigator and evaluated blindly. The rate of apoptosis was quantified with the ApopTag^®^ Peroxidase In Situ Apoptosis Detection kit as previously described (38,39) with a minor modification. Low power observations of retinal sections stained for TUNEL-positive cells with the ApopTag^®^ Plus Fluorescein In Situ Apoptosis Detection kit were made using a Zeiss Axioplan 2 imaging microscope. Fluorescent images were captured with a Photometric CoolSNAP™ HQ CCD camera at a 1392×1040 resolution. Low power images were generated with a 40× acroplan objective using the RS Image™ 1.9.2 software.

### 4-Hydroxynonenal protein adducts and nitrotyrosine ELISA assays

For assessment of HNE adducts, lenses were homogenized in 20 mM PBS, pH 7.4 (1:10, w/v), on ice, and centrifuged at 14,000 × g (4°C, 20 min). Supernatants were used for HNE adducts measurements with the OxiSelect™ HNE-His Adduct ELISA kit (Cell Biolabs, Inc., San Diego, CA). For assessment of NT, lenses were homogenized on ice in RIPA buffer (1:10 w/v) containing 50 mM Tris-HCl, pH 7.2; 150 mM NaCl; 0.1% sodium dodecyl sulfate; 1% NP-40; 5 mM EDTA; 1 mM EGTA; 1% sodium deoxycholate and the protease/phosphatase inhibitors leupeptin (10 μg/ml), aprotinin (20 μg/ml), benzamidine (10 mM), phenylmethylsulfonyl fluoride (1 mM), sodium orthovanadate (1 mM). Homogenates were sonicated (3×5 sec) and centrifuged at 14,000 × g (4°C, 20 min). Supernatants were used for measurements of NT concentrations with the OxiSelect Nitrotyrosine ELISA kit (Cell Biolabs). All the ELISA measurements were performed in accordance with the manufacturer’s instructions. Both HNE adduct and NT concentrations were normalized per mg protein. Protein was measured with the bicinchoninic acid protein assay (Pierce Biotechnology, Rockford, IL).

### Glucose, sorbitol pathway intermediates, and α-glycero- phosphate

Lens glucose, sorbitol, fructose, and α-glycero-phosphate concentrations, and retinal α-glycero-phosphate concentrations were assessed by spectrofluorometric enzymatic methods with hexokinase/glucose 6-phosphate dehydrogenase, sorbitol dehydrogenase, and fructose dehydrogenase as previously described (24,40,41).

### Western blot analysis

Western blot analyses of lens total and phosphorylated p38 MAPK and ERK were performed as previously described (14). Protein bands were visualized with the Amersham ECL western blotting detection reagents and analysis system (GE Healthcare, Buckinghamshire, UK). Membranes were then stripped and reprobed with β-actin antibody to verify equal protein loading. The data were quantified by densitometry (Quantity One 4.5.0 software, Bio-Rad Laboratories, Richmond, CA).

### Cell culture studies

#### Cell preparation

Primary bovine retinal pericyte and endothelial cell cultures were established from fresh cow eyes as previously described (14,42). Passages 4–6 were used for all experiments. Purity of cultures was confirmed by characteristic pericyte and endothelial cell morphology and by the use of specific pericyte (α smooth muscle actin) and endothelial cell (von Willebrand factor) markers. In average, in pericyte experiments, 98.8±1.4% of the isolated cells were identified as pericytes. In endothelial cell experiments, 99.5±1.1% of the isolated cells were identified as endothelial cells.

To dissect effects of glucose and cariporide, pericytes and endothelial cells were cultured in the DMEM-medium containing 20% serum, 100 U/ml penicillin, 100 mg/ml streptomycin, and, for endothelial cells only, 50 μg/ml of endothelial growth supplement. At 50% confluency, pericyte and endothelial cell cultures were transferred to the media i) with 5 mM glucose and without cariporide; ii) with 30 mM glucose and without cariporide, or iii) with 30 mM glucose and with 10 μM cariporide, and cultured for another seven days. At least, three 6-well plates were used per experimental condition. At the end of experiment, the cells were placed on round glass coverslips and coated with gelatin or fibronectin (for pericytes end endothelial cells, respectively).

### Assessment of apoptosis, nitrotyrosine, and poly(ADP-ribose)

At the end of the exposure, the rate of cell death was evaluated with the ApopTag^®^ Plus Fluorescein In Situ Apoptosis Detection kit according to manufacturer’s instructions. Seven to ten images were quantified per experimental condition. The data were calculated as percentage of control i.e., the average value in the cells cultured in 5 mM glucose without cariporide. For assessment of NT and poly(ADP-ribose) by fluorescence immunohistochemistry, coverslips with pericyte or endothelial cells were washed in PBS and fixed in 4% paraformaldehyde for 10 min. Fixed cells were washed in PBS and preincubated with 0.2% Triton X-100 in PBS for 15 min. Coverslips were blocked with 1% BSA containing 10% goat serum for 1 h. Then the cells were treated with either mouse monoclonal anti poly(ADP-ribose) antibody (1:100 dilution) or rabbit polyclonal anti-NT antibody (1:200 dilution). Secondary Alexa Fluor 488 goat anti-mouse or Alexa Fluor 488 goat anti-rabbit antibodies were applied in working dilutions 1:200. The primary antibody was omitted in the negative controls. Coverslips were mounted in Prolong Gold Antifade Reagent and placed on a slide. Images of immunostained cells were captured with a Photometric CoolSNAP™ HQ CCD camera at 1392×1040 resolutions. Fluorescence was quantified using the ImageJ 1.32 software (National Institutes of Health, Bethesda, MD). Seven to ten images were quantified per experimental condition, and the average fluorescence per cell was calculated.

### Statistical analysis

The results are expressed as mean ± SEM. Data were subjected to equality of variance F-test, and then to log transformation, if necessary, before one-way analysis of variance. Where overall significance (P<0.05) was attained, individual between group comparisons were made using the Student-Newman-Keuls multiple range test. Significance was defined at P≤0.05. When between-group variance differences could not be normalized by log transformation (datasets for body weights and plasma glucose), the data were analyzed by the nonparametric Kruskal-Wallis one-way analysis of variance, followed by the Bonferroni/Dunn or Fisher’s PLSD tests for multiple comparisons.

## Results

In the 1-month study, the initial (after STZ administration) non-fasting blood glucose concentrations were increased ~3.4-fold in untreated and cariporide-treated diabetic rats compared with the non-diabetic controls (Table I). Final blood glucose concentrations were 3.7- and 4.1-fold greater in untreated and cariporide-treated diabetic rats than in the non-diabetic control group. Similar levels of glycemia were detected at the initial and final time points in the 3.5-month study, with no differences between diabetic untreated and cariporide-treated groups. NHE-1 inhibition did not affect blood glucose concentrations in non-diabetic rats.

**Figure 1:**
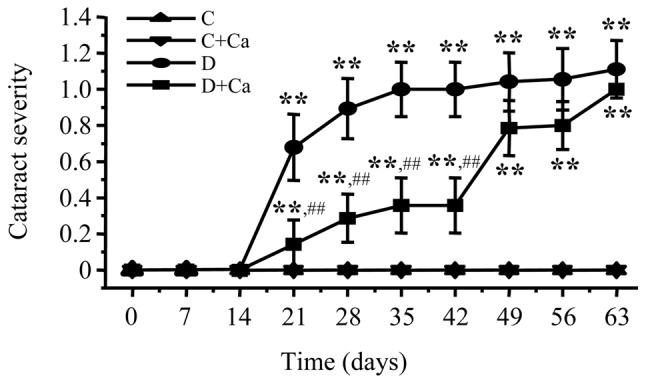
Cataract severities in control and diabetic rats maintained with or without cariporide treatment. C, control group; D, diabetic group; Ca, cariporide. Mean ± SEM, n=10–15/group. ^**^P<0.01 vs. the controls; ^##^P<0.01 vs. the untreated diabetic group.

All the rats had clear lenses during the first two weeks of the study after which cataracts started to form in both diabetic groups (Fig. 1). During the next three weeks, cariporide-treated diabetic rats had lower cataract scores compared with the untreated diabetic group (0.133±0.091 vs. 0.667±0.130; 0.267±0.118 vs. 0.867±0.115; and 0.333±0.126 vs. 0.967±0.102, P<0.01 for all the comparisons). This indicates that NHE-1 inhibition delays, although does not completely prevent, diabetic cataract formation. This trend was maintained during the next three weeks, but the cataract scores did not differ significantly between the untreated and cariporide-treated diabetic groups. Throughout the study, no cataractous changes were recorded in cariporide-treated non-diabetic rats.

Concentrations of α-glycerophosphate were increased in both lens (1.58±0.21 vs. 0.252±0.035 μmol/g lens in controls, P<0.01) and retina (0.324±0.042 vs. 0.135±0.024 nmol/mg prot in controls, P<0.01) in rats with 1-month duration of diabetes, indicative of the early diabetes-induced activation of the upper part of glycolysis as well as of the diversion of the excessive glycolytic flux towards formation of glycolysis by-products in both ocular tissues.

**Figure 2:**
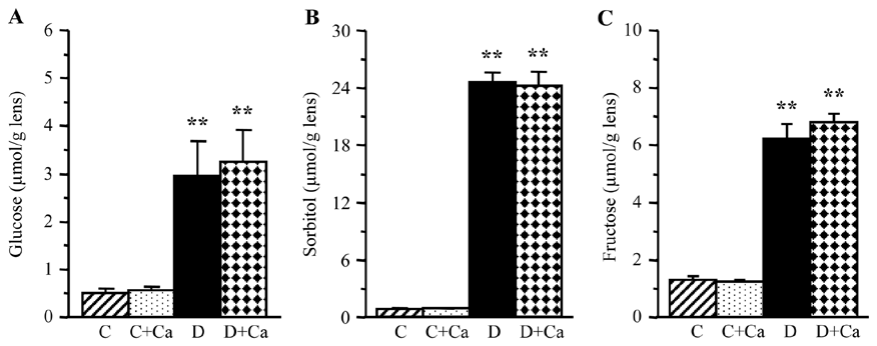
Lens glucose and sorbitol pathway intermediate concentrations in control and diabetic rats maintained with or without cariporide treatment. C, control group; D, diabetic group; Ca, cariporide. Mean ± SEM, n=6/group. ^**^P<0.01 vs. the controls.

Lens glucose, sorbitol, and fructose concentrations were dramatically increased in rats with 1-month duration of diabetes, compared with the corresponding non-diabetic group (Fig. 2). Cariporide treatment did not affect the lens glucose or sorbitol pathway intermediate concentrations in either control or diabetic rats.

**Figure 3:**
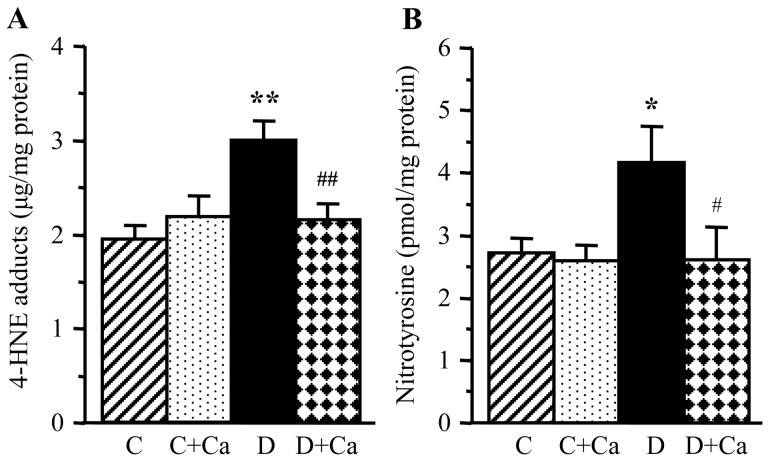
Lens 4-hydroxynonenal adducts and nitrotyrosine concentrations in control and diabetic rats maintained with or without cariporide treatment. C, control group; D, diabetic group; Ca, cariporide; 4-HNE, 4-hydroxynonenal. Mean ± SEM, n=6/group. ^*^P<0.05 and ^**^P<0.01 vs. the controls; ^#^P<0.05 and ^##^P<0.01 vs. the untreated diabetic group.

Lens HNE adducts and NT concentrations were increased by 55 and 53%, respectively, in rats with 1-month duration of diabetes, compared with the non-diabetic controls (Fig. 3). Diabetes-induced HNE adduct and NT accumulation was essentially prevented by cariporide treatment. Cariporide did not reduce either HNE adduct or NT concentrations in the non-diabetic rats.

**Figure 4:**
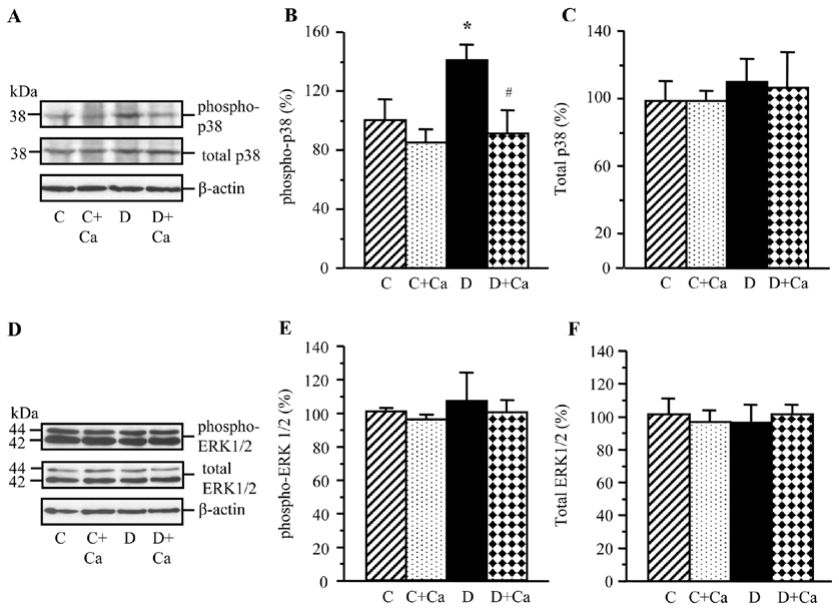
Representative western blot analyses of (A) phosphorylated and total p38 mitogen-activated protein kinase and (D) phosphorylated and total extracellular signal-regulated kinase, and protein contents (densitometry) of (B and C) phosphorylated and total p38 mitogen-activated protein kinase and (E and F) phosphorylated and total extracellular signal-regulated kinase in the lens of control and diabetic rats maintained with or without cariporide treatment. C, control group; D, diabetic group; Ca, cariporide; MAPK, mitogen-activated protein kinase; ERK, extracellular signal-regulated kinase. Mean ± SEM, n=6/group. ^*^P<0.05 vs. the controls; ^#^P<0.05 vs. the untreated diabetic group.

Lens phosphorylated p38 MAPK level was increased in rats with 1-month duration of diabetes, compared with the non-diabetic controls (Fig. 4A and B). Lens total p38 MAPK levels were indistinguishable between the two groups (Fig. 4A and C). Cariporide prevented diabetes-induced increase in the p38 MAPK levels in diabetic rats, without affecting p38 MAPK phopshorylation in the corresponding non-diabetic group. Cariporide did not affect total p38 MAPK levels in either non-diabetic or diabetic rats.

Lens total and phosphorylated ERK levels were similar in the non-diabetic rats and rats with 1-mo duration of diabetes (Fig. 4D–F). Cariporide did not affect total ERK level or ERK phosphorylation in either non-diabetic or diabetic rats.

**Figure 5:**
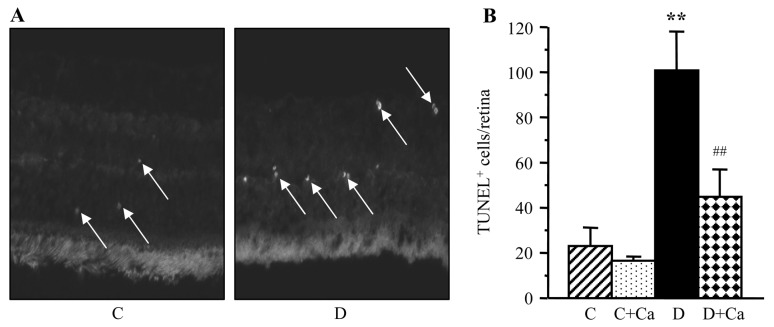
(A) Representative microphotographs of TUNEL-positive cells (TUNEL fluorescence is shown by arrows) in the retinal sections of control and diabetic rats. Magnification, ×40. (B) TUNEL-positive cell counts per flat-mounted retina in control and diabetic rats maintained with and without cariporide treatment. C, control group; D, diabetic group; Ca, cariporide. Mean ± SEM, n=10/group. ^**^P<0.01 vs. the controls; ^##^P<0.01 vs. the untreated diabetic group.

Representative pictures of TUNEL-positive cells (TUNEL fluorescence) in the retinal sections of control and diabetic rats are shown in Fig. 5A. The number of TUNEL-positive nuclei per flat-mounted retina was increased 4.4-fold in rats with 3.5-month duration of diabetes, compared with the non-diabetic controls (Fig. 5B). This increase was partially, but significantly, prevented by cariporide treatment (P<0.01 vs. untreated diabetic group); however, TUNEL positivity was ~2-fold greater in cariporide-treated diabetic rats, than in the non-diabetic controls.

**Figure 6:**
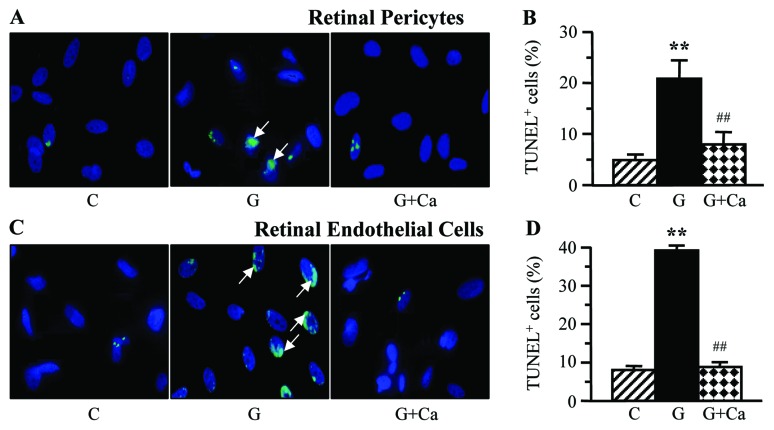
Left, representative microphotographs of TUNEL-positive cells in (A) retinal pericyte and (C) endothelial cell cultures maintained for 7 days i) with 5 mM glucose and without 10 μM cariporide; ii) with 30 mM glucose and without 10 μM cariporide; and iii) with 30 mM glucose and 10 μM cariporide. Magnification, ×100. Blue fluorescence corresponds to 4′,6-diamidino-2-phenylindole-stained nuclei. Right, percentage of TUNEL-positive cells among (B) retinal pericyte and (D) endothelial cell cultured as described above. C, control group (5 mM glucose); G, 30 mM glucose; Ca, cariporide. n=5–8/group. ^**^P<0.01 vs. the cells cultured in 5 mM glucose; ^##^P<0.01 vs. the cells cultured in 30 mM glucose without 10 μM cariporide.

A 7-day exposure to high glucose was associated with augmented cell death in retinal microvascular cells, manifested by more than 4-fold increase in the numbers of TUNEL-positive pericytes (Fig. 6A and B) and endothelial cells (Fig. 6C and D). NHE-1 inhibition essentially (pericytes) or completely (endothelial cells) prevented high glucose-induced increase in TUNEL-positivity.

**Figure 7:**
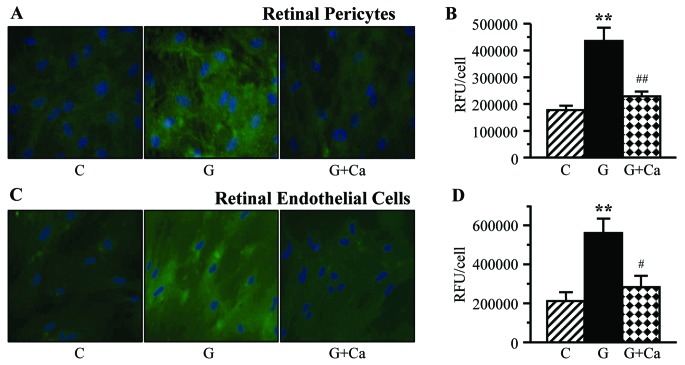
Left, representative microphotographs of nitrotyrosine fluorescence (green) in (A) retinal pericytes and (C) endothelial cell cultures maintained for 7 days i) with 5 mM glucose and without 10 μM cariporide; ii) with 30 mM glucose and without 10 μM cariporide; and iii) with 30 mM glucose and 10 μM cariporide. Magnification, ×100. Blue fluorescence corresponds to 4′,6-diamidino-2-phenylindole-stained nuclei. Right, nitrotyrosine fluorescence (relative fluorescence units/cell) in (B) retinal pericyte and (D) endothelial cell cultures maintained as described above. C, control group (5 mM glucose); G, 30 mM glucose; Ca, cariporide; RFU, relative fluorescence units. n=5–8/group. ^**^P<0.01 vs. cells cultured in 5 mM glucose; ^##^P<0.01 vs. cells cultured in 30 mM glucose without 10 μM cariporide.

Nitrotyrosine fluorescence was increased in high glucose-exposed cultured retinal pericytes (Fig. 7A and B) and endothelial cells (Fig. 7C and D). High glucose-induced nitrative stress was essentially prevented by cariporide in both cell types.

**Figure 8:**
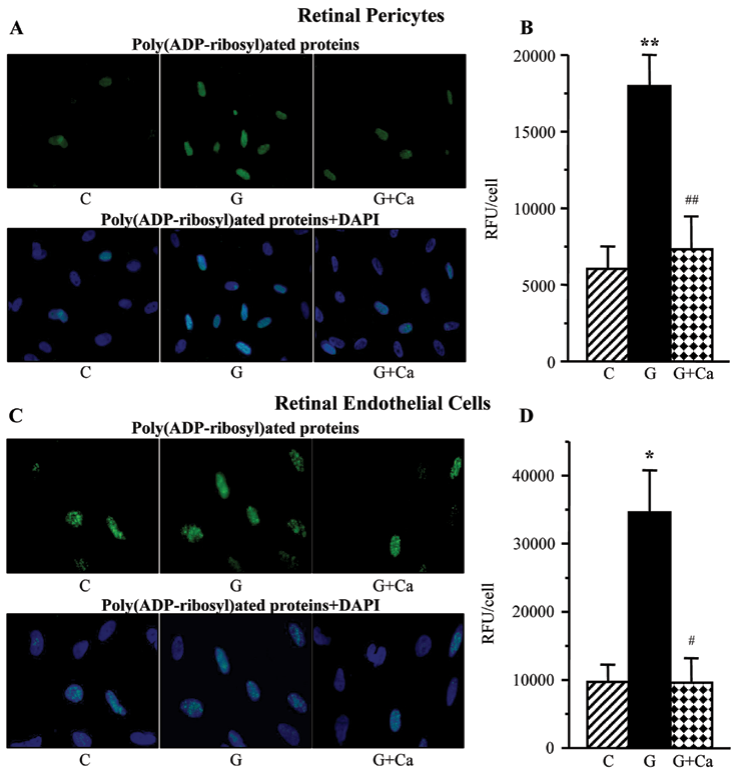
Left, representative microphotographs of poly(ADP-ribose) fluorescence (green) in (A) retinal pericytes and (C) endothelial cell cultures maintained for 7 days i) with 5 mM glucose and without 10 μM cariporide; ii) with 30 mM glucose and without 10 μM cariporide; and iii) with 30 mM glucose and 10 μM cariporide. Magnification, ×100. Blue fluorescence corresponds to 4′,6-diamidino-2-phenylindole-stained nuclei. Right, poly(ADP-ribose) fluorescence (relative fluorescence units/cell) in (B) retinal pericyte and (D) endothelial cell cultures maintained as described above. C, control group (5 mM glucose); G, 30 mM glucose; Ca, cariporide; RFU, relative fluorescence units. n=5–8/group. ^*^P<0.05 and ^**^P<0.01 vs. cells cultured in 5 mM glucose; ^#^P<0.05 and ^##^P<0.01 vs. cells cultured in 30 mM glucose without 10 μM cariporide.

Poly(ADP-ribosyl)ated protein fluorescence was increased in retinal pericytes (Fig. 8A and B) and endothelial cells (Fig. 8C and D) cultured in 30 mM glucose compared with those cultured in 5 mM glucose. Cariporide essentially prevented high glucose-induced accumulation of poly(ADP-ribosyl)ated proteins in both cell types.

## Discussion

In the present study, the NHE-1 inhibitor cariporide delayed, but did not prevent, cataract formation and reduced premature retinal cell death in STZ-diabetic rats. It also counteracted high glucose-induced oxidative-nitrative stress, PARP activation, and apoptosis in cultured retinal pericytes and endothelial cells. These findings have a number of important implications for understanding the mechanisms contributing to diabetic cataractogenesis and early retinopathy as well as the development of new therapeutic approaches.

First, the beneficial effect of NHE-1 inhibition on the lens and retina was not due to alleviation of diabetic hyperglycemia. This is a very important observation because the quality of glycemic control is the most important risk factor for both diabetic ocular complications (43). An early intensification of insulin treatment was associated with about 5-fold reduction of cataract risk in children and adolescents with type 1 diabetes (44). In the UK Prospective Diabetes Study (UKPDS), the tight glycemic control reduced the risk of cataracts in subjects with adult-onset type 2 diabetes (45). Both DCCT/the Epidemiology of Diabetes Interventions and Complications (EDIC) and UKPDS trials identified chronic hyperglycemia as a leading causative factor in the pathogenesis of diabetic retinopathy (46,47). Thus, any agent ameliorating hyperglycemia can be expected to counteract diabetes-induced changes in the lens and retina.

Second, the findings of the present study are consistent with our primary hypothesis that NHE-1 is an important contributor to diabetes- and high glucose-induced oxidative-nitrative stress in the two ocular tissues. Oxidative-nitrative stress resulting from an imbalance between free radical and oxidant production and insufficient upregulation or downregulation of antioxidative defense is present in the lens early during the course of diabetes, and is manifested by accumulation of lipid peroxidation products, malondialdehyde and 4-hydroxyalkenals, depletion of the main biological antioxidant, reduced glutathione, increase in the oxidized-to-reduced glutathione ratio, depletion of other important non-enzymatic antioxidants, ascorbate and taurine, as well as upregulation of antioxidative defense enzyme i.e., superoxide dismutase, glutathione peroxidase, glutathione reductase and glutathione transferase, activities (40,48–52). Consistent with these earlier observations, the present study revealed accumulation of HNE protein adducts and nitrotyrosine in the lens in rats with 1-month duration of STZ-diabetes. A prevention of diabetes-induced increase in both variables by the NHE-1 inhibitor cariporide is in agreement with the multifactorial origin of oxidative-nitrative stress in tissues-sites for diabetic complications including lens [reviewed in (11)]. Increased AR activity and osmotic stress as well as non-enzymatic glycation and glycoxidation have previously been shown to contribute to lenticular oxidative injury through disruption of antioxidative defense mechanisms, activation of NAD(P)H oxidase, as well as generation of free radicals during interactions of advanced glycation end-products with their receptors (11,53,54). Note, that alleviation of oxidative-nitrative stress in cariporide-treated diabetic rats in the present study was not due to reduction in lens glucose and sorbitol pathway intermediate concentrations. Also note, that the role for NHE-1-mediated excessive p38 MAPK phosphorylation in diabetes-induced lens oxidative injury is unclear. A bidirectional relationship was identified between oxidative stress and p38 MAPK phosphorylation in tissue sites for diabetic peripheral neuropathy (21,55). Such studies were never conducted in the lens, although increased p38 and other MAPK phosphorylation was documented previously (56).

Third, the current results generate new knowledge about the role for oxidative-nitrative stress in diabetic cataract formation. In previous studies, natural antioxidants such ascorbate, α-tocopherol, β-carotene, pantethine, the superoxide mimetic tempol, the free radical scavengers amino phosphorothioate (WR-77913) and amifostine (WR2721), the lipid-soluble antioxidant butylated hydroxytoluene and the pyridoindole antioxidant stobadine delayed, but did not completely prevent, diabetic cataract formation (11,13,57,58). Therefore, antioxidants were less effective than aldose reductase inhibitors, the only class of compounds, which completely prevents diabetes-induced cataractogenesis (11). Note, however, that none of the afore-mentioned antioxidant studies presented unequivocable evidence of the complete correction of diabetes-induced lenticular oxidative stress with antioxidant treatment. In the experiments reported herein, cariporide essentially blunted lenticular oxidative-nitrative stress, but, despite this, only delayed diabetic cataract formation. Thus, compared to sorbitol pathway activation and osmotic stress, oxidative-nitrative stress plays a secondary role in diabetes-associated cataractogenesis.

Fourth, previous studies implicated NHE-1 in retinal endothelin production and vasoconstriction at an early stage of diabetes (34). The current findings support and complement these observations suggesting an important contribution of NHE-1 to early diabetic retinopathy. Oxidative-nitrosative stress, manifest in increased lipid peroxidation, accumulation of nitrated and poly(ADP-ribosyl)ated proteins, excessive superoxide production, and downregulation of several antioxidative defense enzymes i.e., superoxide dismutase, glutathione peroxidase, and glutathione reductase, is present in the rat retina early after induction of STZ-diabetes (14,37,59–62). Furthermore, increased generation of reactive oxygen species and accumulation of nitrated and poly(ADP-ribosyl)ated proteins were documented in cultured retinal pericytes shortly after exposure to high glucose (42,63). Oxidative-nitrative stress and PARP activation were identified as the major mechanisms leading to diabetes-induced retinal cell apoptosis, and, at a later stage, to background diabetic retinopathy (13,14,64,65). In the present study, NHE-1 inhibition with cariporide was associated with a significant reduction of premature retinal cell death in STZ-diabetic rats. These *in vivo* findings are in agreement with alleviation of high glucose-induced oxidative-nitrative stress and reduced poly(ADP)-ribosylation and apoptosis in high glucose-exposed pericytes and endothelial cells. Thus, the present animal and *in vitro* data support previous observations (13,14,65), including ours (14), suggesting that diabetes-associated oxidative-nitrative stress and PARP activation play an important role in premature cell death in the whole retina and retinal microvascular cells. Note, that the important role for peroxynitrite injury and poly(ADP)-ribosylation in apoptosis has been demonstrated in animal and cell culture models of several diabetic complications (66–70).

In conclusion, this study is the first to demonstrate the role for NHE-1 in diabetes-induced oxidative-nitrative stress in lens and retina, cataractogenesis, and premature apoptosis in retina and retinal microvascular cells. The findings suggest that NHE-1 may be a therapeutic target for diabetic ocular complications.

## Figures and Tables

**Table I:** Non-fasting blood glucose concentrations (mmol/l) in control and diabetic rats maintained with or without cariporide treatment.

	Groups			
	
Variables	Control	Control + Ca	Diabetic	Diabetic + Ca
1-month study				
Initial	6.73±0.17	6.60±0.11	23.0±0.81^a^	22.9±0.81^a^
Final	6.44±0.5	6.22±0.5	23.8±1.2^a^	25.7±0.83^a^
3.5-month study				
Initial	6.51±0.15	6.42±0.14	21.2±0.61^a^	20.9±0.75^a^
Final	6.64±0.18	6.81±0.19	22.5±0.92^a^	23.5±0.97^a^

Data are expressed as mean ± SEM, n=10–15/group. Ca, cariporide.

a P<0.01 vs. non-diabetic controls.
